# The Power of Auditory-Motor Synchronization in Sports: Enhancing Running Performance by Coupling Cadence with the Right Beats

**DOI:** 10.1371/journal.pone.0070758

**Published:** 2013-08-07

**Authors:** Robert Jan Bood, Marijn Nijssen, John van der Kamp, Melvyn Roerdink

**Affiliations:** 1 MOVE Research Institute Amsterdam, Faculty of Human Movement Sciences, VU University Amsterdam, Amsterdam, the Netherlands; 2 Institute of Human Performance, University of Hong Kong, Hong Kong SAR; University of California, Merced, United States of America

## Abstract

Acoustic stimuli, like music and metronomes, are often used in sports. Adjusting movement tempo to acoustic stimuli (i.e., auditory-motor synchronization) may be beneficial for sports performance. However, music also possesses motivational qualities that may further enhance performance. Our objective was to examine the relative effects of auditory-motor synchronization and the motivational impact of acoustic stimuli on running performance. To this end, 19 participants ran to exhaustion on a treadmill in 1) a control condition without acoustic stimuli, 2) a metronome condition with a sequence of beeps matching participants’ cadence (synchronization), and 3) a music condition with synchronous motivational music matched to participants’ cadence (synchronization+motivation). Conditions were counterbalanced and measurements were taken on separate days. As expected, time to exhaustion was significantly longer with acoustic stimuli than without. Unexpectedly, however, time to exhaustion did not differ between metronome and motivational music conditions, despite differences in motivational quality. Motivational music slightly reduced perceived exertion of sub-maximal running intensity and heart rates of (near-)maximal running intensity. The beat of the stimuli –which was most salient during the metronome condition– helped runners to maintain a consistent pace by coupling cadence to the prescribed tempo. Thus, acoustic stimuli may have enhanced running performance because runners *worked harder* as a result of motivational aspects (most pronounced with motivational music) and *more efficiently* as a result of auditory-motor synchronization (most notable with metronome beeps). These findings imply that running to motivational music with a very prominent and consistent beat matched to the runner’s cadence will likely yield optimal effects because it helps to elevate physiological effort at a high perceived exertion, whereas the consistent and correct cadence induced by auditory-motor synchronization helps to optimize running economy.

## Introduction

On February 18^th^ 1998, the Ethiopian athlete Haile Gebrselassie astonished sport spectators when he achieved a world best time of 4∶52.86 min in the 2000 m. Shortly after the race, Gebrselassie indicated that he had coupled his running cadence with the beat of the pop song *Scatman* by the late Scatman John, which was played throughout his race at Birmingham’s National Indoor Arena, UK.

This anecdote of Gebrselassie and Scatman testifies to the fact that rhythmic bodily movements are often coupled with external acoustic stimuli, such as acoustic metronomes and music, a phenomenon known as auditory-motor synchronization [1], [2], [3]. Dancing to music, for example, involves the synchronization of whole-body movements to the beat [1]. Another striking example is our natural tendency to tap our fingers, hands, or feet along to a beat when listening to music [2], [3], [4]. In fact, research demonstrates that even young infants spontaneously sway and wiggle around with rhythmic acoustic stimuli [5], which supports the notion that humans have a predisposition for auditory-motor synchronization [5], [6], [7].

This apparent predisposition for auditory-motor synchronization has led to the exploitation of acoustic rhythms as a potential means to enhance performance in practical settings, including rehabilitation [8], [9], exercise [10], [11], and sports [12], [13]. With respect to the latter, pundits and experts alike consider acoustic rhythms particularly useful in sports that are cyclic in nature, like running, rowing, and cycling. In coxed rowing, for example, the coxswain in the stern coordinates the stroke rate and rhythm for the rowers to follow. Musical tempo also dictates movement rate in popular exercise-to-music classes, like spinning, aerobics, and Zumba. In spinning classes for example, variations in the tempo of the music determine the pedaling rate and thereby the work rate (i.e., music functions as an external pacemaker).

The potential scope of auditory-motor synchronization as a performance enhancement tool has been demonstrated by the recent development of products that help attune musical content to the actual or desired work rate during running, cycling, rowing, and circuit-class training (e.g., [14], [15], [16], [17]) as well as by recent scientific findings in the context of sports or exercises (i.e., [18], [19], [20], [21]). Specifically, Simpson and Karageorghis demonstrated superior performance (i.e., faster times) of 400 m sprints completed in a synchronous music condition in comparison to a no-music control condition [20]. Likewise, Terry and colleagues reported longer times to volitional exhaustion with than without synchronous music in a group of elite triathletes during high-intensity treadmill running [21]; see also Karageorghis et al. for similar effects in treadmill walking [19]. Karageorghis and Terry aptly summarized these insights as follows: “When athletes work in time to music, they often work harder for longer” [13]. Finally, Bacon and colleagues found that cycle ergometry at a fixed pedaling rate was more efficient (i.e., lower oxygen consumption) when performed synchronously with music than when the musical tempo was set slightly slower than a visually controlled, fixed pedaling rate [18]. These findings underscore the potential of synchronous music and auditory-motor synchronization to enhance work-rate, endurance, and efficiency in cyclic sports.

Music not only provides a stimulus for synchronization, but often also possesses motivational qualities that may enhance performance. The body of evidence on the beneficial effects of motivational music in sports and exercise mainly stems from research on the use of asynchronous, background music (i.e., without an explicit synchronization of movements to the beat). Research suggests that motivational music can enhance sports performance, for example, by lifting mood and arousal levels and by dissociation from feelings of pain and fatigue [13]. With respect to the latter, attending to motivational music during low-to-moderate levels of physical exertion typically reduces perceived exertion by roughly 10% [22]. At higher intensities, attending to music does not reduce perceived exertion. That is, at higher intensities music does not appear to influence *what* one feels (i.e., a similarly high perceived exertion) but only *how* one feels it (i.e., it positively shapes the interpretation of exertion symptoms like fatigue and pain; cf. [13]). Interestingly, not all music seems to be equally effective in terms of motivational quality [23]. For example, loud, fast, percussive music with accentuated bass frequencies has stimulative effects, which increase arousal and associated physiological responses (e.g., heart rate). In contrast, soft, slow music has sedative effects (i.e., reducing arousal). Attending to such music during sports may therefore adversely affect performance. Validated, objective methods, including the Brunel Music Rating Inventory 2 (BMRI-2; [24]), have now also been developed to select music that is likely to yield optimal effects for the task at hand.

It is evident that sport and exercise performance can benefit from synchronous motivational music in terms of ergogenic (i.e., work-enhancing), psychophysical (e.g., perceived exertion), and physiological (e.g., heart rate) effects. It remains unclear, however, whether the beneficial effects are primarily mediated by auditory-motor synchronization, by motivational quality of music, or by a combination of both factors. The opening anecdote concerning Gebrselassie and Scatman highlights the potential of auditory-motor synchronization in enhancing running performance, but motivational quality of the pop song Scatman may also play a role. Interestingly, in a comparative study, Terry et al. [21] observed that elite triathletes ran 18.1% and 19.7% longer, respectively, when running in time to motivational and motivationally-neutral music compared to exhaustive treadmill running in a no-music control condition (see also [20]). This observation suggests that the motivational quality of music may be less important than the prominence of the beat of music and the degree to which participants are able to synchronize their movements to this beat [21]. However, the latter was not quantified.

This study aims to examine the relative effects of auditory-motor synchronization and motivational quality of music on running performance. Therefore, participants will perform three running-to-exhaustion conditions on a treadmill: 1) a control condition without music, 2) a metronome condition with a sequence of beeps matching participants’ cadence (synchronization) and 3) a music condition with synchronous motivational music matching participants’ cadence (synchronization+motivation). The effects of condition will be quantified using a set of complementary ergogenic (time to volitional exhaustion), psychophysical (ratings of perceived exertion), physiological (heart rate), and behavioral (cadence consistency) outcome measures. The latter measure is included to assess the degree to which cadence corresponded with the rhythmic acoustic stimuli (i.e., metronome, music), a methodological advancement to quantify auditory-motor synchronization that has not been embraced by past studies on performance-enhancing effects of synchronous music [18], [19], [20], [21]. An increased time to exhaustion is expected for running with acoustic stimuli (i.e., both metronome and motivational music conditions) in comparison with the control condition without acoustic stimuli. This effect is expected because acoustic stimuli with a tempo matching participants’ cadence promote auditory-motor synchronization, which will likely enhance running efficiency through a more consistent cadence. We further expect a longer time to exhaustion for the motivational music condition (synchronization+motivation) than for the metronome condition (synchronization) because music not only provides a stimulus for auditory-motor synchronization but also possesses motivational qualities that may further enhance running performance.

## Methods

### Participants

To establish sample size, a power analysis for a repeated-measures design was conducted using G*Power 3.1.6 (cf. [25]). Based on the effect sizes reported in comparable studies (e.g., 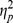 = 0.38 [19], 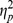 = 0.24 [20]), the analysis indicated that minimally 16 participants for an α of 0.05 and a power of 0.80 would be required. We recruited 19 students (10 males and nine females) from the Faculty of Human Movement Sciences, VU University Amsterdam, to participate in the study (age: 22.5 years of age, range 19–27 years; height: 180 cm, range 163–198 cm; weight: 69 kg, range 50–82 kg). Participants were recreational runners in good physical condition.

### Ethics Statement

The research met all applicable standards for the ethics of experimentation and was approved by the Ethics Committee of the Faculty of Human Movement Sciences of VU University Amsterdam (ECB 2010-02). Participants provided written informed consent prior to the experiment.

### Experimental Procedure and Setup

Participants reported to the laboratory on three occasions at the same time of day, at least 48 h and up to one week apart. In the pre-experimental phase of the first session, participants were acquainted to the laboratory setting and to treadmill running. Subsequently, belt speed was progressively increased every 30 seconds from 9 km/h upwards in a 5-min protocol. At each speed plateau, participants were asked to estimate how long they could maintain running at that speed. The belt speed increments depended on participants’ estimates: +3 km/h for >120 min, +2 km/h for 60–120 min, +1 km/h for 30–60 min, and +0.5 km/h for 15–30 min. The test speed was defined as the speed that participants perceived they would be able continue running at for 7–15 min. On average, test speed was 13.25 km/h (range: 9.5–17.5 km/h). As soon as the test speed was defined, participants completed this 5-min protocol at 9 km/h. Subsequently, participants rested for 15 min until the start of the experiment proper, which was similar for all three sessions.

**Figure 1:**
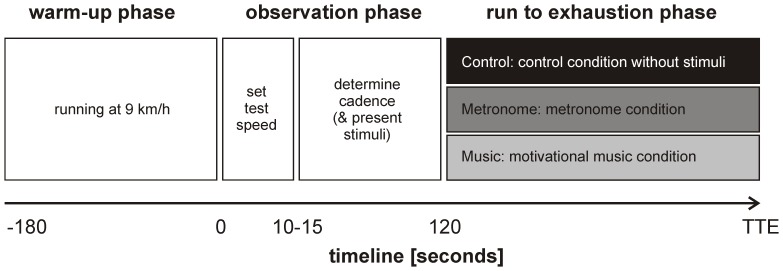
Schematic overview of the experimental design, the experimental phases, and the corresponding timeline. Note that control, metronome, and music conditions are performed in counterbalanced order on separate days, at least 48 h up to one week apart. TTE represents time to volitional exhaustion, which may vary across conditions.

The experiment (see Figure 1) started with a 3-min warm-up phase in which participants ran at 9 km/h. In the subsequent observational phase, belt speed was increased to the test speed and participants’ cadence was determined by counting strides for a 1-min interval in the first two minutes at the test speed. Then, participants were instructed to run to volitional exhaustion in one of three experimental conditions: 1) a control condition without acoustic stimuli, 2) a metronome condition, 3) a motivational music condition. In the latter two conditions, participants were additionally advised to synchronize their steps to the beat of the acoustic stimuli, yet to give priority to running as long as possible. Experimenters did not encourage participants. The test ended when the participant gave a stop signal.

Prior to the motivational music condition, participants were invited to select a song from a motivational music Top 5 (see Table 1). This Top 5 was created as follows. First, a panel of 71 students from VU University Amsterdam were asked to list three artists or bands that produce motivational music for high-intensity sports, like running at high intensity. Then, from each of the top-10 listed performers, two fast songs were selected with a beat per minute (bpm) of 130 bpm or higher following evidence-based recommendations for motivational music (cf. [23]). The motivational quality of the 20 selected songs was rated by an independent panel consisting of four students (mean age: 21.75 years, range 21–22 years) using the 6-item BMRI-2 [24]). The Top 5 comprised the songs that received the highest average BMRI-2 rating score (cf. Table 1).

**Table 1:** Performer and song title of the motivational music Top 5.

Performer	Song title	bpm	BMRI-2	#selected	@bpm
Black EyedPeas	Pump It	153.62	32.50	2	181
The Prodigy	Omen	140.00	31.00	7	173
DJ Tiësto	He’sA Pirate	140.01	30.00	3	178
Red HotChili Peppers	HigherGround	140.78	29.75	5	171
David Guettafeat. Juliet	Do SomethingLove	134.00	29.75	2	158

**bpm** indicates the song’s tempo in beats per minute as verified using Virtual DJ Pro (Atomix Productions), **BMRI-2** scores the song’s motivational quality, **#selected** represents the number of times that the song was selected by participants and **@bpm** indicates the average played tempo of the song to match participants’ cadence.

The tempo of the motivational music and the metronome was matched to the participant’s cadence, with a beat for each footfall to enhance auditory-motor synchronization (cf. [26], [27]). To this end, we used disk-jockey software that enabled us to alter the tempo of the motivational music without changing other aspects of the music (Virtual DJ Pro, Atomix Productions) and a digital metronome (Metronome Plus 2.0.0.1, M & M - Systeme), respectively. Acoustic stimuli were played using a stereo system (Akai QX5690UFX micro music system) at a standardized 80–84 dB volume (verified with Extech HD600), which is loud but still within acceptable noise levels for working environments [28].

Participants performed the three conditions in counterbalanced order on separate occasions. For the exhaustion phase of the experiment we recorded for all three conditions: 1) time to exhaustion (TTE in seconds) using a stopwatch (Oregon Scientific C510 Digital Stopwatch); 2) heart rate every five seconds using a heart rate monitor (Polar S610); 3) rating of perceived exertion (RPE) every minute using Borg’s 15-grade scale positioned at eye-level in front of the treadmill [29]; and 4) cadence on a stride-by-stride basis using a footswitch sensor placed under the left shoe (sampling rate 500 Hz; MA-153 Event Switches, Motion Lab Systems, Baton Rouge, USA).

### Data Preparation, Outcome Measures and Statistical Analysis

We selected the RPE value and the mean heart rate (averaged over the 12 samples) corresponding to the first, central, and final 1-minute segment of the exhaustion phase for each trial for further statistical analyses. The data collected with the footswitch sensor were processed using custom-written Matlab software. After determining event onsets from the footswitch-sensor data, the inverse of event onset intervals was taken to reconstruct cadence time series. To increase the reliability of cadence estimates, they were extracted from *n* moving windows containing 19 intervals (i.e., 20 strides) from which the average cadence was taken (in steps/min). From the so-obtained set of *n* average-cadence observations, we quantified cadence consistency by taking the mean absolute difference between each of the *n* average-cadence observations and the average cadence of the observation phase preceding the exhaustion phase of the experiment (cf. Figure 1). Note that the tempo of the acoustic stimuli was also based on the observed cadence recorded during the observation phase.

The so-obtained TTE, cadence-consistency, RPE, and heart-rate data were first checked for univariate outliers (using an absolute *z*-score criterion of 3.29) as well as normality to ensure that parametric analyses were appropriate. Repeated-measures ANOVAs with the within-subjects factor *Condition* (three levels: control, metronome, music) were performed for TTE and cadence consistency, with post-hoc paired-samples *t*-tests for significant main effects. Repeated-measures ANOVAs with the within-subjects factors *Condition* (three levels) and *Segment* (three levels: first, central, final 60-sec segments of the exhaustion phase) were performed for RPE and heart rate, again with post-hoc paired-samples *t*-tests for significant main or interaction effects. In view of the fact that participants are expected to run longer in the two acoustic stimuli conditions than in the control condition, we furthermore conducted *Condition*×*Time* repeated-measures ANOVAs for RPE and heart-rate data. To this end, RPE and heart-rate values of the metronome and music conditions were anchored to the specific time points of the first, central, and final 60-sec segments of the control condition. Degrees of freedom were adjusted when the sphericity assumption was violated, using either Huynh-Feldt (if Greenhouse-Geisser ε>0.75) or Greenhouse-Geisser (if Greenhouse-Geisser ε<0.75) adjustments [30]. Partial eta squared (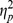) was used to determine effect size. Because effects of conditions on RPE and heart rate are generally subtle in nature, we also determined Cohen’s *d* effect sizes for both acoustic stimuli conditions against the control condition (see Terry et al. [21]).

## Results

The statistical analyses for cadence consistency were based on 16 out of 19 participants as the data from three participants were not available for the ANOVA due to a malfunctioning footswitch sensor in one or more conditions.

### Time to Exhaustion was Longer with than without Acoustic Stimuli

**Figure 2:**
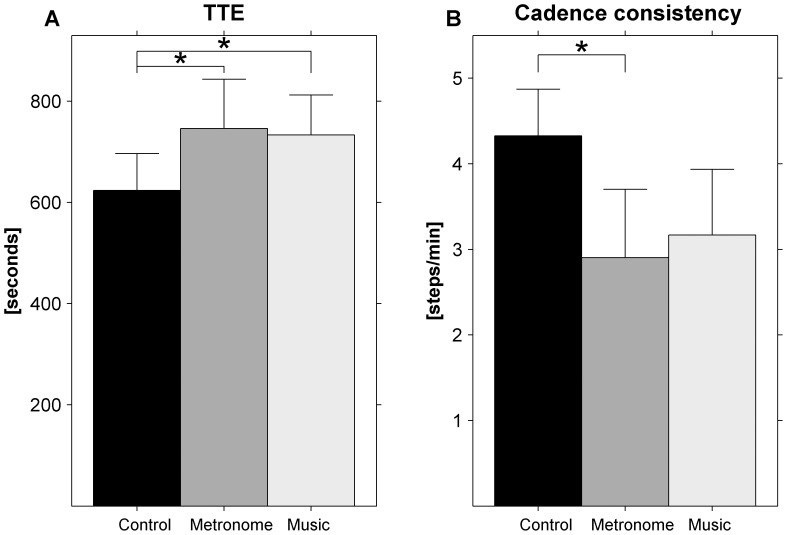
TTE in seconds (A) and cadence consistency in steps/min (B) data of the exhaustion phase for control (black), metronome (dark gray), and motivational music (light gray) conditions. Error bars represent the standard error while asterisks indicate significant differences across conditions.

The time to exhaustion differed significantly across conditions (*F*(2, 36) = 5.05, *p = *0.012, 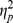 *= *0.219; Figure 2a). Compared to the control condition (TTE = 624 seconds), participants ran significantly longer with acoustic stimuli (metronome: TTE = 746 seconds, *t*(18) = 2.97, *p = *0.008, music: TTE = 733 seconds, *t*(18) = 2.43, *p = *0.026). The time to exhaustion did not differ significantly between metronome and music conditions (*t*(18) = 0.318, *p* = 0.75).

### Cadence was most Consistent in the Metronome Condition

Cadence consistency differed significantly across conditions (*F*(2, 30) = 3.84, *p = *0.033, 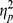 *= *0.204; Figure 2b). Cadence consistency differed significantly between control (4.33 steps/min) and metronome (2.90 steps/min, *t*(15) = 2.30, *p = *0.036) conditions. The difference in cadence consistency between control (4.33 steps/min) and music (3.17 steps/min) conditions was non-significant (*t*(15) = 1.78, *p = *0.095). Cadence consistency did not differ significantly between metronome and music conditions (*t*(15) = 0.876, *p* = 0.39).

### Perceived Exertion and Heart Rate Revealed that Participants Ran to Exhaustion in all Conditions

**Figure 3:**
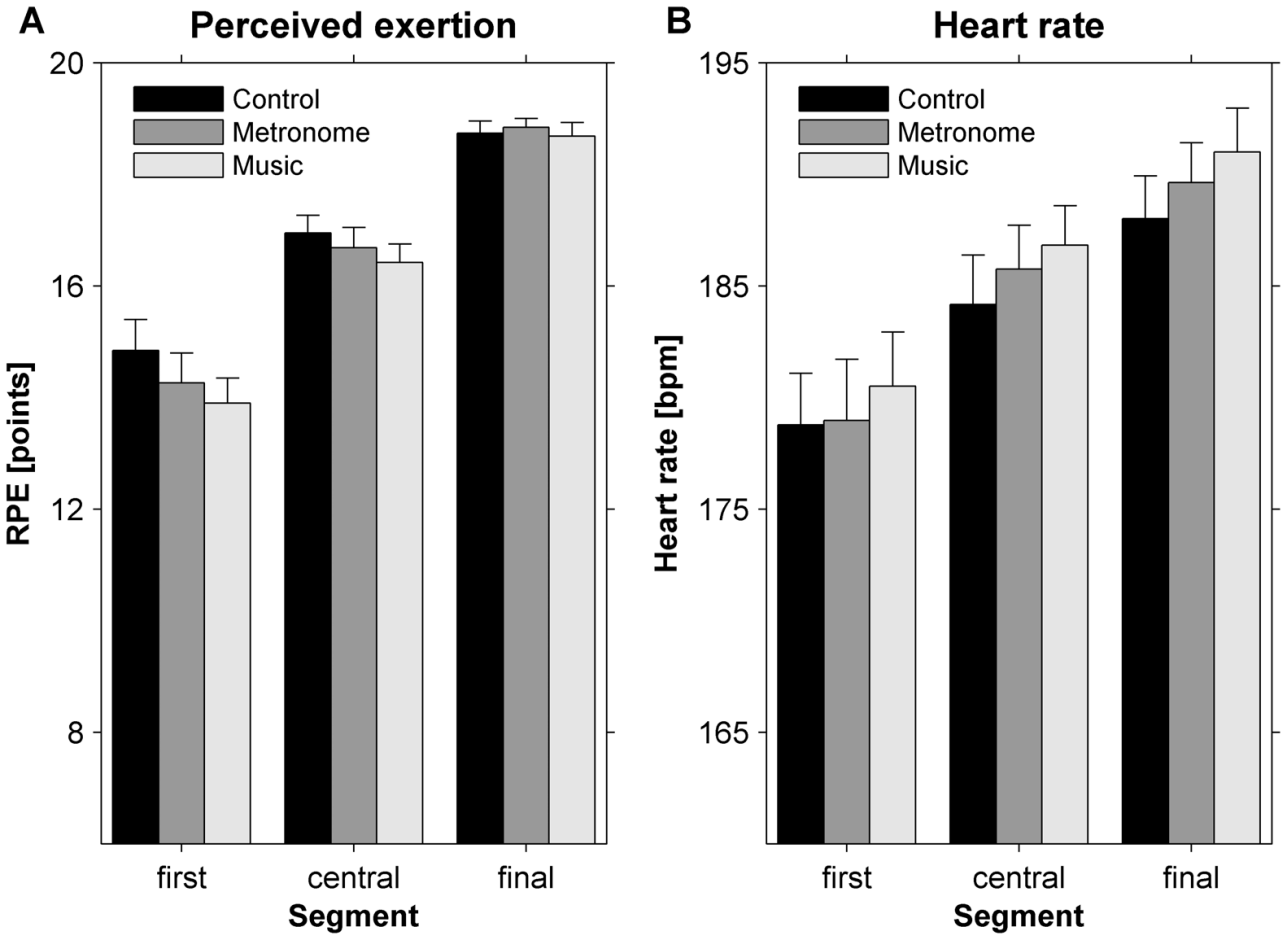
Perceived exertion (A) and heart rate (B) data for the first, central, and final 1-minute segments of the exhaustion phase for control (black), metronome (dark gray), and motivational music (light gray) conditions. Error bars represent the standard error. RPE and heart rate of each segment differed significantly from each other.

A significant main effect of *Segment* was observed for both RPE and heart rate (*F*(1.15, 20.68) = 70.94, *p*<0.001, 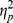 *= *0.798 and *F*(1.09, 19.57) = 51.09, *p*<0.001, 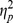 *= *0.739, respectively). Post-hoc analysis for RPE and heart rate revealed significant differences between each segment (all *t*(18)′s>6.58, all *p*′s <0.001; Figure 3). No main or interaction effects involving the *Condition* factor were observed (all *F*′s <1.99, all *p*′s>0.151, all 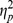
′s <0.100). Nevertheless, close inspection of Figure 3 suggests that RPE and heart rate tend to vary somewhat across conditions. Indeed, small-to-moderate reductions in RPE were observed for the first (metronome: *d* = −0.30; music: *d* = −0.45) and the central segment (metronome: *d* = −0.21; music: *d* = −0.43), but not for the final segment (metronome: *d* = 0.13; music: *d* = −0.10; see also Figure 3a). Furthermore, small-to-moderate increments in heart rate were observed for the final (metronome: *d* = 0.28; music: *d* = 0.49) and the central segment (metronome: *d* = 0.17; music: *d* = 0.35), but not for the first segment (metronome: *d = *0.02; music: *d* = 0.20; see also Figure 3b).

When anchoring RPE values of the metronome and music conditions to the time points of the three segments of the control condition, we observed a significant main effect for *Condition* (*F*(1.88, 33.91) = 7.708, *p* = 0.002, 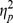 *= *0.300), with post-hoc analyses revealing that RPE was significantly lower in the music condition (15.9) than in the control condition (17.1; *t*(18) = 1.281, *p* = 0.02). RPE for the metronome condition (16.4) did not differ significantly from control and music conditions (*t*(18) = 0.754, *p* = 0.08 and *t*(18) = 0.526, *p* = 0.165, respectively). Furthermore, a significant main effect for *Time* was observed (*F*(2, 36) = 70.079, *p*<0.001, 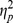 *= *0.796); similar to the main analyses, RPE values increased significantly as a function of time (all *t*(18)′s>2.018, *p*′s <0.001). The *Condition* × *Time* interaction was non-significant (*F*(2.26, 40.66) = 1.014, *p* = 0.380, 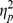 *= *0.053). Anchoring heart-rate values of metronome and music conditions to the time points of the three segments of the control condition again resulted in a significant main effect *Time* (*F*(1.04, 18.66) = 44.61, *p*<0.001, 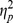 *= *0.713; heart rate increased significantly as a function of time [all *t*(18)′s>3.25, *p*′s <0.001]). Main and interaction effects involving the factor *Condition* were non-significant (*F*(2, 36) = 0.814, *p* = 0.451, 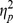 *= *0.043; *F*(2.00, 35.99) = 1.946, *p* = 0.158, 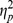 *= *0.098, respectively).

## Discussion

The current experiment sought to examine the relative effects of the motivational quality of music and auditory-motor synchronization with the beat on running performance. Participants ran to exhaustion on a treadmill without acoustic stimuli (control condition), with a metronome beat matched to participants’ cadence (synchronization), and with motivational music with a beat that matched participants’ cadence (synchronization+motivation). Time to exhaustion differed significantly across conditions. In line with our hypothesis we found that the time to exhaustion was longer in the metronome and motivational music conditions than in the control condition without acoustic stimulation. Specifically, participants ran approximately two minutes longer with acoustic stimuli in comparison with a control condition (Figure 2a). Comparable effects have recently been reported for elite triathletes who were instructed to run to self-selected synchronous motivational music, synchronous oudeterous music (i.e., motivationally neutral music) and in a no-music control condition ([21] see also [19] for a similar study on treadmill walking to exhaustion). Time to exhaustion was longer with than without music, regardless of its motivational quality. In combination, these results suggest that the motivational quality of music may be less important than the prominence of the beat of music, which allows participants to synchronize their pace to the prescribed tempo of the acoustic stimulus. We additionally expected differential effects of the two acoustic stimuli conditions on time to exhaustion in view of the clear difference in motivational quality between metronome and motivational-music conditions. However, this was not the case (Figure 2a). In the following, we will discuss motivation, synchronization, and dissociation effects associated with the two types of acoustic stimuli on psychophysical (e.g., perceived exertion), physiological (e.g., heart rate), and behavioral (e.g., cadence consistency) outcome measures to explain why we did not find the expected superior time to exhaustion for the synchronous motivational music condition (i.e., synchronization+motivation).

### Psychophysical and Physiological outcome Measures are Affected more by Motivational Music than by Metronomes

With regard to psychophysical outcome measures, Terry and colleagues [21] recently reported evidence for lower perceived exertion during sub-maximal running to neutral and motivational music compared to a control condition without music (Cohen’s *d*-values ranging from 0.19 to 0.39). In the present study, ratings of perceived exertion varied significantly with *Segment* (Figure 3a). Similar to Terry et al. [21], we found indications for small-to-moderate reductions in perceived exertion with acoustic stimuli, particularly for the first 1-minute segment of sub-maximal running, and most prominently, for motivational music (i.e., *d* = −0.45). This segment-dependent effect is in agreement with previous studies, which indicate that motivational music reduced perceived exertion for sub-maximal intensities [19], [31] but not for maximal intensities [22], [32], [33]. We further observed that the physiological outcome measure heart rate was affected by acoustic stimuli. In the final, near-maximal segment (Figure 3b), small-to-moderate increments in heart rate were observed with acoustic stimuli, particularly during the final 1-minute segment and again most prominently for the motivational music condition (i.e., *d* = 0.49).

The combined effects of acoustic stimuli on psychophysical and physiological outcome measures suggest that for a given sub-maximal heart rate (as observed for the first segment) the presence of acoustic stimuli lowered participants’ perceived exertion, a finding in line with Terry and colleagues [21]. Interestingly, for a given (near-)maximal perceived exertion (as observed for the final segment) the presence of acoustic stimuli may have helped to elevate the attainable physiological load (i.e., higher heart rate). With acoustic stimuli, and in particular with motivational music, participants appeared to be able to work at a higher intensity (higher heart rate at the final segment) for longer (increased TTE) at a comparably high-level rating of perceived exertion. When RPE values were anchored to the time points of the three segments of the control condition, we indeed found that attending to acoustic stimuli reduced perceived exertion considerably, again most prominently for the motivational music condition. This effect was present at all three time points of the running-to-exhaustion phase. Consistent with previous studies on the control of physiological strain during strenuous endurance exercises (e.g., [34]), these findings suggest that athletes actively regulate their relative physiological strain, that is, relative to their perceived exertion. Assuming similar auditory-motor synchronization effects for both acoustic pacing conditions, one would therefore expect a superior effect of the synchronous motivational music condition on the time to exhaustion (allowing participants to work harder for longer) because motivational music had a stronger effect on physiological and psychophysical outcome measures than the metronome given the evident difference in motivational quality between metronome beeps and motivational music. This was, however, not the case, implying that other performance enhancing factors were involved that were - as discussed in the following two paragraphs - seemingly: 1) more effective for the metronome condition than for the motivational music condition (i.e., synchronization); and 2) also effective for the metronome condition (i.e., dissociation).

### Cadence is more Consistent for Running with a Metronome than for Running with Motivational Music

For the metronome condition, running cadence was most consistent, as evidenced by a significant difference in cadence consistency between control and metronome conditions. Enhanced cadence consistency may help to improve running economy because energy loss associated with accelerations and decelerations in cadence is reduced. Moreover, the runner is forced to maintain the *right* cadence, that is, the cadence that was adopted by the runner in the pre-experimental phase prior to the exhaustion phase of the trial (see Figure 1), which is likely to be near to the optimal running cadence for the imposed running speed.

**Figure 4:**
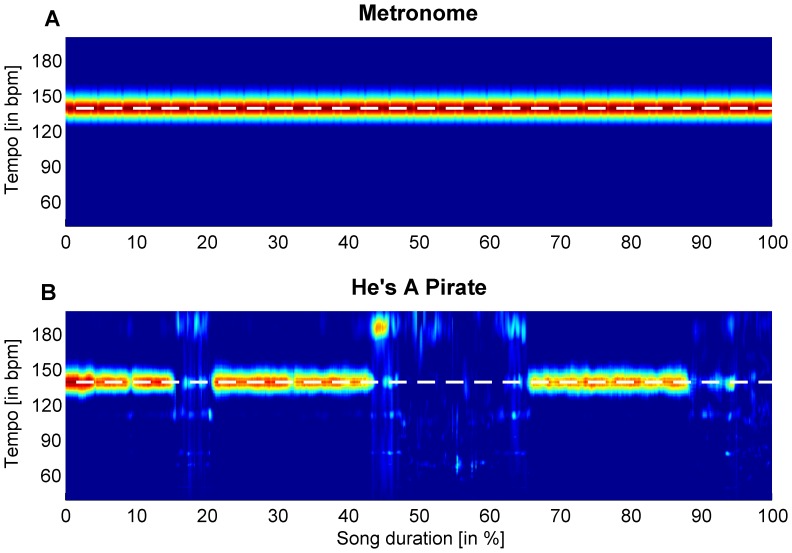
Spectrograms of acoustic stimuli, both at 140 bpm, as indicated by the dashed white line. The ‘hotter’ the color, the more prominent the beat. (A) Spectrogram of the metronome, showing a constant beat. (B) Spectrogram of the motivational music track ‘He’s A Pirate’ by DJ Tiësto, where the tempo is not as constant, readily apparent, and prominent throughout the song as the beat in the metronome.

Recently, Bacon et al. [18] reported that a cyclic exercise was performed more efficiently when executed synchronously with music than when the musical tempo was set slightly slower than the cyclical movement rate. Unlike the current study, in which we quantified cadence consistency, Bacon et al. [18] did not register the revolutions per minute. Hence it is unclear whether the enhanced cycling efficiency with synchronous music was due to: 1) differences in the consistency of the movement relative to the beat of the music, 2) differences in movement tempo between slower, synchronous, and faster music conditions, or 3) an interaction between these two factors. As far as we know, the current study is the first to demonstrate that movement consistency was significantly affected by acoustic stimuli. Specifically, we showed that the type of acoustic stimuli affected auditory-motor synchronization. That is, running cadence was most consistent in the presence of a metronome beat that was matched to the runners’ preferred cadence at the imposed speed. In contrast, the difference in cadence consistency between control and motivational music conditions only tended toward significance (*p* = 0.095). This is presumably due to the retrospective observation that the beat was not as constant, apparent, and prominent throughout the song as the beat in a sequence of metronome beeps (see Figure 4). Hence, from a research point of view (but see also the Practical Recommendations section), a limitation in the study may be that, despite tempo matching, the motivational music only provided a sub-optimal template for auditory-motor synchronization. As a consequence, running cadence was more variable. It is important to note, however, that this is an inherent feature of music.

### Dissociation through Auditory-Motor Synchronization

Another well-known way in which motivational music may influence running performance is by narrowing attention, specifically by diverting it from running-induced feelings of fatigue and discomfort [13], [35]. Focusing attention on motivational music for its distraction effect is a known and effective dissociation technique, especially in athletes who prefer to be distracted from physiological signals in shaping their performance (i.e., so-called dissociators [36]). The idea behind dissociation is that people can only process a limited amount of information at any given time. Thus, dissociation induced by focusing on motivational music, including its lyrical content, may alter the perception of effort, allowing runners to work harder for longer [13]. Likewise, the very act of auditory-motor synchronization may also contribute to dissociation because auditory-motor synchronization is known to be an attention demanding process [3]. Peper and colleagues [37], for example, recently employed a stimulus-response reaction-time task to quantify the attentional costs of walking with and without acoustic pacing. They showed that reaction times were significantly longer with acoustic pacing, emphasizing the elevated attentional demands for auditory-motor synchronization (see also [38]). The same may be true for running with acoustic stimulation with a beat that matches the participant’s preferred cadence, regardless of the motivational quality of the stimuli (viz. motivational music vs. metronome). This is an area of research that deserves more attention, especially for its profound practical implications in enhancing performance in sport, exercise, and rehabilitation settings.

### Practical Recommendations

Given that both the motivational quality of music and the beat of acoustic stimuli appear to have an effect on running performance, it is of practical importance to optimize both of these aspects. Indeed, this is exactly what we did in the motivational music and metronome conditions of the present study. With regard to the former, following existing research findings, we selected loud, fast, percussive music with accentuated bass frequencies [23]. We employed the validated and objective BMRI-2 [24] to optimize the selection of motivational music, resulting in a Top 5 (see Table 1) based on the motivational music preferences for high-intensity sports of a large student panel. A limitation of this procedure is that we could not guarantee that participants actually liked the selected music, which might have diminished the motivational effect of the selected music. However, we chose this procedure because it allowed us to select songs with at least 130 bpm, thus adhering to evidence-based recommendations for motivational music (cf. [23]). Note that in previous research, the music selection in the study of Terry et al. (online appendix in [21]) may have been suboptimal because in both neutral and motivational music conditions, the beats per minute of the music were adjusted to the stride frequency of the participant (i.e., a beat for one step per stride), which is much slower than recommended for motivational music [23]. In contrast, in the present study, we adjusted the beats per minute of the music to the step frequency of the participant (i.e., a beat for both steps per stride), resulting in a much faster beat, as recommended for motivational music [23].

A second benefit of adjusting the tempo of the acoustic stimuli to the cadence of the runner was that it created optimal conditions for auditory-motor synchronization in general [3], [25], [39] and for bipedal locomotion in particular [9], [26], [40], where the beat should pace both footfalls per stride [26] and match the preferred cadence [9], [40]. For the metronome condition, these recommendations for optimal auditory-motor synchronization was easily fulfilled by creating a regular beat sequence. In contrast, in the motivational music condition, we successfully modified the average tempo of the song. However, this modification did not necessarily mean that the resultant beat was constant, readily apparent, and prominent throughout the song due to, for example, the fact that musical intermezzos and other tempo irregularities are inherent to music (cf. Figure 4). Thus, even though the beats per minute of the motivational music were adjusted to match the preferred cadence of the runner, the music still may have provided a less effective reference for synchronization than the metronome. That is, in the latter condition, the beat was always prominent, constant, and readily apparent throughout the running-to-exhaustion phase of the experiment.

### Conclusions

Motivational quality of music positively influences perceived exertion of sub-maximal running intensity as well as heart rates for a (near-)maximal running intensity, which may enhance running performance because it allows runners to *work harder*. Next to motivational quality, the music’s beat may help runners to maintain a consistent pace if they couple their cadence to the prescribed tempo of the acoustic stimulus, which may enhance running performance by helping athletes *work more efficiently*. Therefore, running to motivational music with a very prominent and consistent beat that is matched to the runner’s preferred cadence will likely yield optimal effects because it helps elevate physiological strain at a very high perceived exertion, while the consistent and correct cadence induced by auditory-motor synchronization helps to facilitate running economy. Motivational music with the right beat may therefore help runners to work harder *and* more efficiently, which is likely to enhance their running performance.

## References

[1] Miura A, Kudo K, Ohtsuki T, Kanehisa H (2011) Coordination modes in sensorimotor synchronization of whole-body movement: a study of street dancers and non-dancers. Hum Mov Sci 30: 1260–1271. 21802159 10.1016/j.humov.2010.08.006

[2] Large EW (2000) On synchronizing movements to music. Hum Mov Sci 19: 527–566.

[3] Repp B (2005) Sensorimotor synchronization: A review of the tapping literature. Psychon Bull Rev 12: 969–992. 16615317 10.3758/bf03206433

[4] Wilson EM, Davey NJ (2002) Musical beat influences corticospinal drive to ankle flexor and extensor muscles in man. Int J Psychophysiol 44: 177–184. 11909649 10.1016/s0167-8760(01)00203-3

[5] Zentner M, Eerola T (2010) Rhythmic engagement with music in infancy. Proc Natl Acad Sci U S A 107: 5768–5773. 20231438 10.1073/pnas.1000121107 PMC2851927

[6] Patel A (2008) Music, Language, and the Brain. New York, NY: Oxford University Press.

[7] Zatorre RJ, Chen JL, Penhune VB (2007) When the brain plays music: Auditory-motor interactions in music perception and production. Nat Rev Neurosci 8: 547–558. 17585307 10.1038/nrn2152

[8] Thaut MH (2008) Rhythm, Music, and the Brain: Scientific Foundations and Clinical Applications. New York, NY: Routledge.

[9] Roerdink M, Bank PJM, Peper CE, Beek PJ (2011) Walking to the beat of different drums: Practical implications for the use of acoustic rhythms in gait rehabilitation. Gait Posture 33: 690–694. 21454077 10.1016/j.gaitpost.2011.03.001

[10] Karageorghis CI, Priest DL (2012) Music in the exercise domain: a review and synthesis (Part I). Int Rev Sport Exerc Psychol 5: 44–66. 22577472 10.1080/1750984X.2011.631026 PMC3339578

[11] Karageorghis CI, Priest DL (2012) Music in the exercise domain: a review and synthesis (Part II). Int Rev Sport Exerc Psychol 5: 67–84. 22577473 10.1080/1750984X.2011.631027 PMC3339577

[12] Bishop DT (2010) ‘Boom Boom How’: Optimising performance with music. Sport Exerc Psychol Rev 6: 35–47.

[13] Karageorghis CI, Terry PC (2011) Inside sport psychology. Champaign, IL: Human Kinetics.

[14] Moens B, van Noorden L, Leman M (2010) D-Jogger: syncing music with walking. Available: http://smcnetwork.org/files/proceedings/2010/66.pdf. Accessed 2013 Jan 08.

[15] van der Vlist B, Bartneck C, Mäueler S (2011) moBeat: Using interactive music to guide and motivate users during aerobic exercising. Appl Psychophysiol Biofeedback 36: 135–145. 21380562 10.1007/s10484-011-9149-y PMC3084945

[16] Wijnalda G, Pauws S, Vignoli F, Stuckenschmidt H (2005) A personalized music system for motivation in sport performance. IEEE Pervasive Comput 4: 26–32.

[17] Westerink JH, Claassen A, Schuurmans T, IJsselsteijn W, de Kort Y, et al.. (2011) Runners’ experience of implicit coaching through music. In: Westerink J, Krans M, Ouwerkerk M, editors. Sensing emotions: The impact of context on experience measurements. Philips Research Book Series 12, 121–134. Springer Book Archives, ISBN 978-90-481-3258-4.

[18] Bacon CJ, Myers TR, Karageorghis CI (2012) Effect of music-movement synchrony on exercise oxygen consumption. J Sports Med Phys Fitness 52: 359–365. 22828457

[19] Karageorghis CI, Mouzourides D, Priest DL, Sasso T, Morrish D, et al (2009) Psychophysical and ergogenic effects of synchronous music during treadmill walking. J Sport Exerc Psychol 31: 18–36. 19325186 10.1123/jsep.31.1.18

[20] Simpson SD, Karageorghis CI (2006) The effects of synchronous music on 400-m sprint performance. J Sports Sci 24: 1095–1102. 17115524 10.1080/02640410500432789

[21] Terry PC, Karageorghis CI, Saha AM, D’Auria S (2012) Effects of synchronous music on treadmill running among elite triathletes. J Sci Med Sport 15: 52–57. 21803652 10.1016/j.jsams.2011.06.003

[22] Boutcher SH, Trenske M (1990) The effects of sensory deprivation and music on perceived exertion and affect during exercise. J Sport Exerc Psychol 12: 167–176.

[23] Karageorghis CI, Terry PC, Lane AM, Bishop DT, Priest DL (2012) The BASES Expert Statement on use of music in exercise. J Sports Sci 30: 953–956. 22512537 10.1080/02640414.2012.676665

[24] Karageorghis CI, Priest DL, Terry PC, Chatzisarantis NLD, Lane AM (2006) Development and validation of an instrument to assess the motivational qualities of music in exercise: The Brunel Music Rating Inventory-2. J Sports Sci 24: 899–909. 16815785 10.1080/02640410500298107

[25] Faul F, Erdfelder E, Lang AG, Buchner A (2007) G*Power 3: a flexible statistical power analysis program for the social, behavioral, and biomedical sciences. Behav Res Methods 39: 175–191. 17695343 10.3758/bf03193146

[26] Kudo K, Park H, Kay BA, Turvey MT (2006) Environmental coupling modulates the attractors of rhythmic coordination. J Exp Psychol Hum Percept Perform 32: 599–609. 16822126 10.1037/0096-1523.32.3.599

[27] Roerdink M, Lamoth CJC, van Kordelaar J, Elich P, Konijnenbelt M, et al (2009) Rhythm perturbations in acoustically paced treadmill walking after stroke. Neurorehabil Neural Repair 23: 668–678. 19307435 10.1177/1545968309332879

[28] Williams W (2005) Noise exposure levels from personal stereo use. Int J Audiol 44: 231–236. 16011051 10.1080/14992020500057673

[29] Borg G (1998) Borg’s perceived exertion and pain scales. Champaign, IL: Human Kinetics.

[30] Girden ER (1992) ANOVA: repeated measures. Newbury Park, CA: Sage Publications.

[31] Nethery VM (2002) Competition between internal and external sources of information during exercise: Influence on RPE and the impact of the exercise load. J Sports Med Phys Fitness 42: 172–178. 12032412

[32] Schwartz SE, Fernhall B, Plowman SA (1990) Effects of music on exercise performance. J Cardiopulm Rehabil 10: 312–316.

[33] Tenenbaum G, Lidor R, Lavyan N, Morrow K, Tonnel S, et al (2004) The effect of music type on running perseverance and coping with effort sensations. Psychol Sport Exerc 5: 89–109.

[34] Esteve-Lanao J, Lucia A, de Koning JJ, Foster C (2008) How do humans control physiological strain during strenuous endurance exercise? PLoS ONE doi:10.1371/journal.pone.0002943. 10.1371/journal.pone.0002943 PMC2491903 18698405

[35] Karageorghis CI, Terry PC (1997) The psychophysical effects of music in sport and exercise: A review. J Sport Behav 20: 54–68.

[36] Morgan WP, Pollock ML (1977) Psychological characterization of the elite distance runner. Ann NY Acad Sci 301: 382–403. 270929 10.1111/j.1749-6632.1977.tb38215.x

[37] Peper CE, Oorthuizen JK, Roerdink M (2012) Attentional demands of cued walking in healthy young and elderly adults. Gait Posture 36: 378–382. 22608701 10.1016/j.gaitpost.2012.03.032

[38] Lamoth CJC, Roerdink M, Beek PJ (2007) Acoustically-paced treadmill walking requires more attention than unpaced treadmill walking in healthy young adults. Gait Posture 26: S96–S97.

[39] Roerdink M, Ridderikhoff A, Peper CE, Beek PJ (2012) Informational and neuromuscular contributions to anchoring in rhythmic wrist cycling. Ann Biomed Eng, In press. 10.1007/s10439-012-0680-7 PMC3701797 23099793

[40] Roerdink M, Lamoth CJC, Kwakkel G, van Wieringen PCW, Beek PJ (2007) Gait coordination after stroke: Benefits of acoustically paced treadmill walking. Phys Ther 87: 1009–1022. 17553922 10.2522/ptj.20050394

